# Household food insecurity and health in a high-migration area in rural Honduras

**DOI:** 10.1016/j.ssmph.2021.100885

**Published:** 2021-08-03

**Authors:** Sanjeev Kumar, Nicholas A. Christakis, Rafael Pérez-Escamilla

**Affiliations:** a Department of Health Policy and Management, Yale School of Public Health, Yale University, New Haven, CT, USA; b Human Nature Lab, Department of Sociology, Yale University, New Haven, CT, USA; c Department of Social and Behavioral Sciences, Yale School of Public Health, Yale University, New Haven, CT, 06510, USA

**Keywords:** Honduras, Food insecurity, Migration, Gender, Depression, Mental health, Measure of overall health, Wellbeing, Logistic regression models

## Abstract

Household food insecurity (HFI) is a significant problem in the developing world. Relationships between HFI and nutrition, physical growth, and development have been elucidated; less is known about the non-nutritional impacts among individuals living in rural areas in low-income countries. The aim of this study was to determine if HFI is a risk factor for suboptimal mental health and overall health in rural Honduras. In a population of 24,696 adults with 176 isolated villages in western Honduras, we collected data on household food insecurity and physical and mental health outcome measures. Using logistic regression with and without adjusting for village and household level unobservables invariant across individual respondents, we show that females (OR: 1.11, p <0.01)), indigenous people (OR: 2:00, p < 0.01), and those planning to migrate (OR: 1.24, p <0.01) have higher odds of experiencing food insecurity. The risks of food insecurity and poor health were mitigated among respondents living where they were born and having multi-generations of relatives living in the same village—a measure of the opportunity and availability of social networks. Living in a food insecure compared to a food secure household was associated with 77 percent higher odds of being depressed, 35 percent higher odds of low overall mental health, and 20 percent higher odds for low overall health.

## Highlights

• Article identifies the potential causes and consequences of household food insecurity in a high migration area in rural Honduras.
• Study addresses food insecurity in the context of gender issues and social connectedness in a marginalized rural community in Honduras.
• More individuals reported stress and anxiety about being food insecure than to actually running out of food.
• Household food insecurity is a significant risk factor for both mental and overall health in a vulnerable population in the 'Dry Corrider' area in Honduras.

## 1 Introduction

Household food insecurity (HFI) is a significant problem, harming individuals and whole economies (Elgar et al., 2021; Horton & Hoddinott, 2014; Kristof, 2019; Stuart, 2017; Weaver & Hadley, 2009). Ending hunger and achieving food security is one of the 2030 sustainable development goals (SDG's) set by the United Nations in 2015 (Stuart, 2017); however, less than 1% of global assistance goes to addressing undernutrition (Kristof, 2019). Research suggests that the return to the investment in nutrition and food security continues to remain very high, with approximately 30 USD return for every dollar spent on nutrition (Horton & Hoddinott, 2014). Relationships between HFI and nutrition, physiological growth, and development have been elucidated; however, less is known about the non-nutritional impacts, especially for those living in rural areas in developing and fragile economies like Honduras (Weaver & Hadley, 2009). Recent studies analyzing data from over 100 countries collected through the Gallup World Poll using FAO's Food Insecurity Experience Scale (FIES), have consistently shown that food insecurity is a major risk factors for the mental health and overall well-being of individuals, and this relationship has been documented in both rural and urban areas (Elgar et al., 2021; Jones, 2017).

Food security (FS) is determined by: 1) the availability of enough quality of food within the community/country at all times; 2) adequate economic and physical access at the household and individual level; 3) the utilization of these food within the family, as well as access to clean water and good sanitation conditions allowing the safe preparation of food (FAO et al., 2017). The first experience-based household food insecurity (HFI) scale was developed in the United States. The so called US Household Food Security Survey Module eventually led to the Latin American and Caribbean Food Security Scale (ELCSA) and the FAO's Food Insecurity Experience Scale (FIES) currently being used to help track the Sustainable Development Goal (SDG) 2 globally (FAO et al., 2017).

A recent study conducted with FIES data from 134 countries identified five common socio-demographic risk factors for HFI: 1) low level of education, 2) less social capital, 3) weak social networks, 4) low income, and 5) being unemployed (Smith et al., 2016). In 2016, the overall prevalence of severe HFI in the world was 9.3%, with the highest percentage in Sub-Saharan Africa at 31% (FAO et al., 2017). HFI has been associated with poor physical health and lower self-reported well-being across countries with different levels of economic development (Frongillo et al., 2017). This is expected as HFI can affect both the quality and quantity of food consumed and is predicted by poverty. Indeed, a study conducted with nationally representative data from Mexico documented a dose-response relationship between HFI severity level and stunting (Shamah-Levy et al., 2017). Ironically, studies conducted in diverse countries have also consistently found an association between HFI and obesity in women (Schlussel et al., 2013). Furthermore, the double burden of malnutrition (defined as a stunted child living in households with overweight/obese mothers) has also been found to be independently associated with HFI in Brazil (Mahmudiono et al., 2018) and Indonesia (Gubert et al., 2017).

A mixed methods study with 434 mothers from Nicaragua identified an association between HFI and maternal distress, and social support from parents mediated this relationship. Interestingly, the qualitative results from this study showed that participants considered only their close family as being a source of social support for them, since fear of gossip and embarrassment kept them from looking for help outside of their near family network (Piperata et al., 2016). A study in Northern Uganda with 403 pregnant women found an independent relationship between HFI and the severity of depressive symptoms, and this relationship was moderated by social support (Natamba et al., 2017; Perkins et al., 2018). A study from Iran also found the buffering effect of social support against mental helath problems among individuals living in households with different levels of food insecurity (Sadiddin et al., 2019).

A scoping review of studies focusing on high income countries found that there is enough evidence backing up the relationship between HFI and mental health in women to support the formulation of policies and programs to address this worrisome public health concern (Maynard et al., 2018). HFI has consistently been found to negatively impact the health of individuals, specially increasing the risk for the development of chronic diseases. Data from a cross-sectional representative survey conducted in the U.S. found HFI associated with increased levels of C-reactive protein, which is a biomarker reflecting inflammation that has been correlated with chronic disease risk (Gowda et al., 2012; Gubert et al., 2017). HFI has also been associated with type 2 diabetes in the U.S. and Canada, and consistent with the obesity findings, this relationship was stronger among women (Laraia, 2013). An analysis of data from the 2005–2012 National Health and Nutrition Examination Survey (NHANES) documented an association between HFI and cardiometabolic conditions such as diabetes mellitus, hypertension, coronary heart disease, congestive heart failure, and obesity (Berkowitz et al., 2017). Another nationally representative study from the U.S. found an association between hypertension and HFI (Seligman et al., 2009). In Mexico, data from a nationally representative survey showed that HFI was associated with a higher risk of self-reported type 2 diabetes among women, especially among those experiencing moderate HFI (Perez-Escamilla et al., 2014).

HFI is considered a major life stressor that has consistently been shown to be negatively associated with mental health. Indeed, NHANES findings identified a positive association between HFI and depressive symptoms among adults with diabetes and prediabetes (Montgomery et al., 2017). A U.S. community study found an association between HFI and depression symptoms among low-income Latinos with type 2 diabetes (Kollannoor-Samuel et al., 2011). Another U.S. study found that among people with diabetes, HFI was associated with increased stress levels which in turn were associated with increased HbA1C levels or poor blood glucose control (Walker et al., 2018). In another U.S. study, HFI was found to be associated with a higher risk of depression, and a SNAP program participation modified this relationship (Leung et al., 2015). A study conducted in Quito, Ecuador, in low-income neighborhoods found that moderate to severe HFI was associated with a higher risk of self-reporting health status as being fair/poor. This study also found that moderate to severe HFI was associated with an increased risk of reporting depression and stress symptoms (Weigel et al., 2016). An analysis of data from the 2014 Gallup World Poll from 149 countries found that HFI was associated with poor self-reported mental health following a dose-response pattern as a function of the level of severity of HFI (Jones, 2017).

Even though there is consistent evidence for an association between HFI and poor physical and mental health outcomes, there is limited evidence from local studies, especially in the rural areas located in low-income countries characterized by higher levels of migration, to understand if and how these relationships work.

Though Honduras has made significant progress in its efforts to improve the health of its population, there is still much that remains to be done (Shakya et al., 2016). Honduras has lately been in the news because of the number of Honduran migrants apprehended at the southern United States border. A recent report suggests that their number has surged from 47,900 in 2017 to 205,039 in just the first nine months of 2019 (Border, 2019). The prevailing narrative around corruption in Honduras is often offered as a reason, but corruption is a longstanding phenomenon (Chetwynd et al., 2003; LaFeber, 1993; Nazario, 2019). Migrant caravans are not just fleeing poverty and violence, walking thousands of miles to find ways to get inside the US (Nazario, 2019). Many of these families are experiencing food insecurity.

However, migration is a very complex and costly decision. Before the final decision to migrate, individuals and families make plans, save money, activate their social networks, etc. Hence, it is important to build a comprehensive framework to identify factors associated with health and food insecurity, and migration in Honduras (Ben-Davies et al., 2014). The existing literature has mainly explored if food insecurity leads to migration (Sadiddin et al., 2019; Smith & Wesselbaum, 2020), but it is important to acknowledge that the opposite may also be true; i.e. that the intention to migrate could itself be a source of financial distress leading to food insecurity as the process of migration is relatively expensive and migrants need to save money to be able to successfully migrate.

Research undertaken to understand more comprehensively the relationship between food insecurity and overall wellbeing, including mental health outcomes, is of direct importance to global healthcare professionals and to policy-makers. Specifically for Honduras, it can inform the design of policy interventions to mitigate the negative impact of food insecurity for the health of vulnerable populations, including those living in rural areas. Thus, the specific objectives of this study are to (a) identify risk factors for HFI and (b) examine the association between HFI and risk of poor mental and physical health and well-being in a large community study in Copán Honduras.

## 2 Conceptual model: Theoretical framework and possible mechanisms

HFI can lead to both macro- and micro-nutrient deficiencies. HFI, especially during early childhood and adolescence, may have long-lasting consequences. It has been shown that both in the animals as well as with human models, subjects under duress take high risks (Bendahan et al., 2017; Friedman et al., 2017). Food insecurity may increase the practice of risky-health behaviors including smoking, binge drinking, and the consumption of licit and illicit psychoactive substances. These coping strategies, in turn, can lead to or exacerbate existing diseases and health conditions. Jones (Jones, 2017) and Gundersen and Ziliak (Gundersen & Ziliak, 2015) delineate the stress response mechanism to connect HFI with mental health and depression. Uncertainty over the ability to maintain access to food in the future, a key dimension of the HFI construct, may contribute to depression and anxiety. In existing research in LMICs, the focus has largely been on severe HFI, emphasizing the experiences of women rather than men (Jones, 2017; Weaver & Hadley, 2009), and often excluding vulnerable rural populations—a contribution that we make to this strand of literature.

The potential stress and anxiety mechanisms discussed above by Gundersen and Ziliak provide a framework to think about the role that HFI plays in overall mental and general health status, including the onset of chronic diseases (Ben-Davies et al., 2014). While those who are poor are more likely to experience HFI, it is not obvious what aspects of poverty-related mediators can be addressed, which is essential knowledge to understand how to improve food security.

Poverty leads to stress, and stress impairs the ability to make sound economic decisions, leading to more poverty. On the other hand, it may also be the case that a food secure poor household might be less likely to be depressed and hence less likely to remain poor in the longer term. Additionally, empirical evidence has shown that depression contributes negatively to labor market outcomes. One of the salient features of depression is pessimistic beliefs about the efficacy of one's efforts: a depressed person attributes a negative shock to his or her abilities, while a positive shock is attributed to luck (de Quidt & Haushofer, 2017). Such misattribution is likely to affect one's propensity to experience the world and thus lessens exploratory behavior and narrows the choice set that one faces, leading to suboptimal decisions.

## 3 Context

Honduras, with a population of almost 10 million, is one of the poorest countries in Latin America and has one of the world's highest murder rates (CIA, 2018). Most residents live in the mountainous western half of the country, and the economy suffers from an extraordinarily unequal distribution of income as well as high unemployment as well as underemployment (CIA, 2018).

Honduras falls in the ‘Dry Corridor’ area in Central America, an area particularly susceptible to irregular and long-lasting droughts. The 2018 Global Nutrition Report documents that in Honduras, around 58 percent of children living in the Dry Corridor are undernourished and have stunted growth as a result (Report, 2018). Compounding the challenge of food insecurity, Honduras is suffering from the double burden of malnutrition and obesity. About one-third (32%) of the population live on less than $3.20/day, while 16% live on less than $1.90/day. FAOSTAT reports that 15% of Hondurans suffer from undernourishment (Faostat, 2018). Stunting for children under 5 remains high at 22.6% for the whole of the country; it is inordinately high at 42% for the bottom 20% of the population according to household-level income. In rural areas, stunting prevalence is 14.6%. Anemia among pregnant and non-pregnant women of reproductive age is reported to be high, at almost 20% (Report, 2018).

## 4 Study sample

Data for our study were collected from 176 villages located in 4 municipalities from the department of Copan. Honduras is divided into 18 departments, each of which has a governor. The department of Copan, which is in the western part of Honduras, on the border with Guatemala, is an area of over 200 square miles of rugged mountainous terrain with an estimated population of 92,000 people who live in small villages on about two dollars per day.

This study is an analysis of baseline data collected as part of a randomized controlled trial (RCT) of a novel social network targeting techniques in order to explore how social network dynamics affect the uptake, diffusion, and group-level normative reinforcement of key reproductive, maternal, neonatal and child health (RMNCH) behaviors and attitudes in rural Honduras (Shakya et al., 2016). Given the high level of adolescent pregnancy, those aged 12 and above were surveyed. The total number of residents in this age range in a census was 30,818, residing in 10,826 unique household units (with 1697 units being single-member households). The final sample ended up including 24,818 respondents residing in 10,015 households.

Table 1 presents the descriptive statistics for the study population. About 42% were male, with a mean age of 33 years. About 12% of the respondents reported indigenous status—most belonging to the Maya Chorti ethnicity. 51.13% report being Catholic; 32.33% Protestant; 0.08% Mormon; 16.45% no religion. Only 3.0% of respondents reported having secondary or more than secondary level of schooling. 35% of the respondents report low overall health, while 32% categorize themselves as having poor mental health. Almost 60% of respondents reported living in the village where they were born. A total of 95.4% report no plan to migrate. Among those who report plans to leave their village, most report that they plan to go to other villages within the same department. Only 1.27% report having plans to leave for another country.

**Table 1:** Sample characteristics, Copán, Honduras.

Variable	N	Mean	Std. Dev.	Min	Max
**Household**	30818			1	10826
**Village**	30818			0	176
**4-Scale HFI**	24818	1.74	1.04	1	4
**HFI Dummy**	24818	0.45	0.50	0	1
**Severe HFI**	24818	0.14	0.35	0	1
**3-Scale HFI**	24818	1.59	0.73	1	3
**Male**	24812	0.42	0.49	0	1
**Age**	24812	32.83	17.16	12	93
**Indigenous**	24798	0.12	0.32	0	1
**No Religion**	24812	0.16	0.37	0	1
**Both Parents in the Village**	24797	0.39	0.49	0	1
**Not Married**	24812	0.35	0.48	0	1
**Widowed**	24812	0.04	0.20	0	1
**Overall Health**	24809	2.24	1.06	0	4
Low Overall Health Dummy	24812	0.44	0.50	0	1
**Mental Health**	24797	2.13	1.11	0	4
**Low Mental Health Dummy**	24812	0.39	0.49	0	1
**Depression**	24810	0.53	0.75	0	3
**Depression Dummy**	24812	0.42	0.49	0	1
**No Education**	24809	0.22	0.41	0	1
**Grade 1**–**6**	24809	0.69	0.46	0	1
**Some Secondary Education**	24809	0.07	0.25	0	1
**Secondary or More**	24809	0.03	0.17	0	1
**Plan to Migrate**	24787	0.05	0.21	0	1
**Born in the Village**	24812	0.60	0.49	0	1
**Wealth Index 1 (Poorest)**	24396	0.22	0.41	0	1
**Wealth Index 2**	24396	0.20	0.40	0	1
**Wealth Index 3**	24396	0.21	0.41	0	1
**Wealth Index 4 (Richer)**	24396	0.20	0.40	0	1

While for the Copan region around 4.6% reported intention to migrate, in our study intention to migrate ranged from 0 to 14% across villages. Among those who were between 15 and 50 years old, 5.1% reported that they intended to migrate, largely within Honduras, and for the older age group, only 3 percent reported this intention. Among the four municipalities, the intention ot migrate ranged form 7% in the Jeronimo municipality to 3.8% in the Copan Ruinas municipality.

### 4.1 Measures of food insecurity

Food insecurity refers to the inability to afford enough food for an active, healthy life (Seligman et al., 2009). Measuring food (in)security is challenging as most food insecurity is seasonal or regular but aperiodic, i.e., associated with temporary unemployment, episodes of ill health, or other recurring adverse events. Moreover, even the expectation of being food insecure in the future could be a source of anxiety and stress, and one needs to factor in such anxiety-related to the availability of food. For our study, a set of two questions was used to measure HFI. These two questions were derived from the Latin American and Caribbean Food Security Scale (ELCSA) (Pérez-Escamilla et al., 2009); (Pérez-Escamilla et al., 2007). Only two out of the original 14 questions were included because of survey duration limitations. The exact wording was modified based on whether the respondent was an adult or a minor. Each of the questions represents an end of the spectrum of the level of severity of HFI. As in previous studies (Hager et al., 2010), we verified the validity of using a reduced number of items.

Two questions described below were used to create a categorical food (in)security indicator—food secure, mild food insecurity, moderate food insecurity, and severe food insecurity:

(1) In the past 3 months, for lack of money or other resources, did you ever worry that your household would run out of food?
(2) In the past 3 months, for lack of money or other resources, did your household ever run out of food? Those who did not affirm any of the two questions were considered food secure; those affirming only the first or second question as mildly food insecure; and those affirming both questions as severely food insecure.

### 4.2 Health measures

Three self-reported health measures—depression, overall mental health, and overall health—are used as the outcome measures. The first questions were processed from Patient Health Questionnaire-2 (PHQ-2) ((Kroenke et al., 2003); (Thibault & Steiner, 2004); (Arroll et al., 2010)) derived from the first two items of the PHQ-9. These two questions make it easier to routinize inquiry about the most prevalent and treatable mental disorders in primary care. These two questions were as follows: (1) Are you feeling down, depressed, or hopeless? Response options were: Not at all, More than the half days, Several days, Nearly everyday, Don't Know, Refused. (2) Now, thinking of your mental health, including stress, depression, and emotional problems, how would you rate your overall mental health? (3) Generally, you would say that your health is. For the last two questions, response options were: Excellent, Very Good, Good, Fair, Poor, Don't Know, Refused.

## 5 Data analysis

### 5.1 Variable(s) selection

The Causal Inference Framework (CIF) developed by Pearl (1995) was used to make decisions about the inclusion of independent variables in the final model, keeping in mind their potential for being a confounder, a mediator, or a collider (Pearl, 1995). A confounder is a causal antecedent of both treatment (HFI) and outcome (Health). A mediator mediates the interaction between treatment and outcome. A collider is a variable that is affected by both the treatment (HFI) as well as outcome variables (Health). Adjusting for a collider induces association (introduces bias) even for the treatment and the outcome variables that are independent. The main objective of this paper is largely descriptive, as there is a dearth of information on the relationship between HFI and health outcomes in highly marginalized and geographically isolated communities. The (confounding) predetermined variables—age, gender, marital status, birthplace—were included as they were assumed to affect both the treatment variable—HFI—as well as the health outcome variables.

Given the necessity of food and nutrition in the biological functioning of humans, we assumed that food insecurity would affect education, wealth, and even religious affiliation. A food-insecure household is assumed to be more likely to seek social network support and hence to be approached by or approach an organized religious organization to seek help to address their HFI. Moreover, in our models, the health outcome variables, in turn, were assumed to determine these variables, thus turning these variables as outcomes of both HFI and health outcomes—i.e., colliders; thus, adjusting for these variables could bias the estimates (Pearl & Mackenzie, 2018).

Many villages and household-level unobserved characteristics invariant across individuals—village level inequality, social dynamics, intrahousehold bargaining power—might not only be the confounders, but some of them could potentially be colliders for the relationship between HFI and health outcomes. We present results with and without including these fixed effects in the econometric analyses.

### 5.2 Econometric models

In order to probe the risk factors that could potentially shed light on HFI, we focused first on the correlates of HFI using logistic regression. HFI is an ordinal measure, and it is important to factor in the possibility that the degree of severity of HFI food insecurity could have a differential effect in terms of their risky health behaviors and social relationships. Though we used the Generalized Ordered Logistic Models to investigate these possible associations, we chose to report odds ratios results from the simple logistic models for ease of interpretation. In order to use the logistic regression framework, we dichotomized the health outcome variables. HFI was modeled as a 3-scale measure ranging from food secure to severely food insecure.

First, we adjusted for a set of standard demographic and socio-economic variables to understand the risk factors associated with any or the severe form of HFI. We subsequently adjusted for the village- and household-level fixed effects to control for the nature of selection on unobservables at village or household level.

(1) $$logit\left(P_{H_{ihv}}\right)=\alpha_{hv}+X_{ihv}\gamma_{1}+\varepsilon_{ihv}$$

$P_{H_{ihv}}$ is the odds of experiencing HFI for individual ‘*i*’ from household ‘*h*’ living in village ‘*v*’. X is the set of explanatory variables mentioned above. ε captures all the unobserved factors. In the second phase of the analysis (captured by Eqn. (2) below), we used the main explanatory variable of interest, HFI, in the regression. We used the coefficient estimates from specification based on the variables selection approach described above as well as with the household-level fixed effects to infer the extent of the selection on unobservable variables:

(2) $$logit\left(P_{Y_{ihv}}\right)=\alpha_{hv}+HFI_{ihv}+X_{ihv}\gamma_{1}+\varepsilon_{ihv}\;$$

$P_{Y_{ipv}}$ is the odds ratio different measures of heath outcomes. We estimated the coefficients from the village- and household-fixed effects as a robustness measure and also to understand the nature of mediation by the village or household invariant factors.

We also ran separate analyses for the respondents from Copan Ruinas municipality—the largest of the four municipalities in the survey (results not reported in the paper). This was done to investigate if the uncovered relationships for the full sample continued to hold even for the Copan Ruinas sample; this would inform whether the relationships were less likely to hold because of some unobserved confounders at the municipality or region levels—for example, efficiency of municipal administrators.

## 6 Results

### 6.1 Descriptive findings

Table 1 shows that in the study area, 54.34% reported being food secure while 34% report mild to moderate food insecurity, and 14% reported experiencing severe food insecurity. For any form of HFI along measures of economic distress, 74% of those in the most economic distress category (ED-4) reported some form of food insecurity, while for those in the least economic distress (ED-1), only around 10% reported any form of HFI. The corresponding numbers for ED-2 and ED-3 are 31% and 58%.

In all four municipalities, the respondents reported to have been much more likely to be worried about food (43%) than actually running out of food (16%). Panel 3 of Table 2 shows that 44% of females reported being worried about food insecurity while 17% report their households running out of food. Respondents affiliated with institutionalized religion report a higher level of food insecurity. The last two rows report the corresponding measures for indigenous versus non-indigenous population: 63% reported worrying and 25% report running out of food—the highest incidence of food insecurity among the demographic markers.

**Table 2:** Disaggregated food security measures, Copán, Honduras.

						
	**Worry About Food Security**			**Food Ran Out**		
**I. Municipality**	**N**	**Mean**	**Std. Dev.**	**N**	**Mean**	**Std. Dev.**
**Cabanas**	3785	0.44	0.50	3785	0.13	0.34
**Copan Ruinas**	10662	0.45	0.50	10662	0.16	0.37
**San Jeronimo**	2392	0.38	0.49	2393	0.15	0.36
**Santa Rita**	7926	0.43	0.49	7923	0.16	0.36
**II. Demographic**	N	Mean	Std. Dev.	N	Mean	Std. Dev.
**Male**	10516	0.42	0.49	10514	0.13	0.34
**Female**	14307	0.44	0.50	14307	0.17	0.38
**Religion**	20743	0.44	0.50	20741	0.16	0.36
**No Religion**	4080	0.42	0.49	4080	0.15	0.36
**Total**	24823	0.43	0.50	24821	0.16	0.36
**Non-Indigenous**	21913	0.41	0.49	21909	0.14	0.35
**Indigenous**	2896	0.63	0.48	2897	0.25	0.43
**III. Age Categories**	**N**	**Mean**	**Std. Dev.**	**N**	**Mean**	**Std. Dev.**
**12–19**	7126	0.29	0.46	7124	0.10	0.30
**20–35**	8412	0.42	0.49	8411	0.14	0.35
**36–50**	4982	0.54	0.50	4983	0.19	0.39
**51–65**	2904	0.57	0.49	2904	0.24	0.43
**66 and above**	1396	0.55	0.50	1396	0.22	0.42
**Total**	24820	0.43	0.50	24818	0.16	0.36

Panel 4 of Table 2 reports the food insecurity measures across different age groups. It is the age group 51–65 that felt most food insecure. A far lower percentage of those in the age group 11–19 reported any form of FI.

### 6.2 Risk factors for food insecurity

In the most parsimonious models, where we adjusted for only demographic and wealth levels—age, gender, ethnicity, widow-status, religion, marital status, social support, and wealth quintiles—women were more likely to report HFI: 12 percent higher odds for any type of HFI and 45 higher odds for the severe HFI both highly significant (p < 0.01) (see Table 3).

**Table 3:** Multivariate logistic regression: Risk factors for household food insecurity in Copán, Honduras.

	Food Insecurity (0,1)		Severe Food Insecurity (0,1)	
	1	2	1	2
Female	1.117***	1.117***	1.440***	1.448***
	(0.032)	(0.033)	(0.060)	(0.061)
Age	1.015***	1.017***	1.013***	1.017***
	(0.001)	(0.001)	(0.001)	(0.002)
Indigenous	2.510***	2.032***	2.005***	1.266***
	(0.110)	(0.117)	(0.103)	(0.089)
Widowed	1.024	1.007	1.257***	1.224**
	(0.075)	(0.076)	(0.104)	(0.104)
Plan to Migrate	1.249***	1.247***	1.535***	1.519***
	(0.082)	(0.084)	(0.136)	(0.138)
No Religion	1.054	1.044	1.087	1.079
	(0.040)	(0.041)	(0.058)	(0.060)
Both Parents in the Village	0.915***	0.905***	0.897**	0.883**
	(0.031)	(0.032)	(0.045)	(0.046)
No Education	3.529***	2.858***	7.266***	5.242***
	(0.366)	(0.306)	(1.688)	(1.236)
Grade 1-6	2.332***	2.030***	4.061***	3.274***
	(0.231)	(0.206)	(0.933)	(0.760)
Some Secondary Education	1.343***	1.276**	1.512	1.397
	(0.152)	(0.147)	(0.390)	(0.363)
Not Married	0.818***	0.832***	1.030	1.075
	(0.030)	(0.031)	(0.054)	(0.058)
Born in the Village	1.006	1.006	0.981	0.958
	(0.031)	(0.033)	(0.041)	(0.043)
Wealth Index 2 (Poorer)	0.963	0.945	0.918	0.915
	(0.046)	(0.047)	(0.058)	(0.060)
Wealth Index 3	0.881***	0.901**	0.811***	0.853**
	(0.041)	(0.044)	(0.051)	(0.056)
Wealth Index 4	0.830***	0.865***	0.701***	0.764***
	(0.038)	(0.041)	(0.044)	(0.050)
Wealth Index 5 (Richest)	0.762***	0.794***	0.655***	0.706***
	(0.034)	(0.037)	(0.040)	(0.045)
Constant	0.218***		0.022***	
	(0.026)		(0.005)	
Observations	23,680	23,680	23,680	23,680
Number of Village		176		176

Note: Robust standard errors clustered at the village level in parentheses; ***p < 0.01, **p < 0.05, *p < 0.1; Column.1s: Odd ratios from an unadjusted logistic regression; Column 2s: Adjusted odd ratios after with village level fixed effects.

Respondents categorizing themselves as indigenous were found to be more food insecure. Those reporting indigenous status were two times greater odds of being food insecure, statistically significant at 1%, after adjusting for the pre-determined confounders mentioned above. The likelihood of severe HFI for indigenous people was 1.27 times greater odds (p < 0.01) (see Table 3).

Widowhood was found to be strongly associated with the severe form of HFI: 1.25 greater odds of being severely food insecure (p < 0.01); however, the uncovered association with any form of food insecurity was found to be negligible. A similar relationship was uncovered for the religion variable.

Respondents who did not identify themselves with any religion (Catholic, Pprotestant, Mormons, or any other religion) were no more likely than the religious ones to experience food insecurity. In the parsimonious specification, respondents with no religion had only 1.05 times higher odds of being food insecure and 1.11 times higher odds of experiencing the severe form of HFI. However, after adjustment for household-level unobservable variables, the relationship became statistically insignificant.

Education and wealth both were also found to be associated with HFI. The noteworthy finding for the education variables was the steep gradient in the likelihood of being food insecure: those with no education reported 2.85 times higher odds (p < 0.01), those with only primary education 2.03 times (p < 0.01), and those with some secondary 1.27 times greater odds (p < 0.01) of being food insecure in comparison to that those with secondary or higher-level education in any given village. The corresponding numbers for being severely food insecure were (5.24, 3.27, and 1.40), all with p < 0.01. In comparison to the poorest wealth quintile, the relationship between the wealth quintile and HFI showed a monotonic relationship: being in the poorer quintile was not found to be associated with any greater odds of being food insecure while being in the middle, the 3rd quintile, had 12% lower odds, being in the 4th quintile with 17% lower odds, and being in the richest quintile with 24% lower odds of experiencing any HFI in comparison to those in the poorest quintile, and all the odds ratios were found significant at 1%. The same pattern was found in the case of severe form of HFI.

The variable capturing intention to migrate turned out to be statistically significant and the magnitude of the estimated coefficient was high enough to be of economic relevance (except for the specification with household-level fixed effects). Respondents who reported their intention to migrate had 1.25 (1.53) times higher odds (p < 0.01) of any form (severe form) of HFI.

The variable capturing the possibility of harnessing social support—the presence of both parents in the village—turned out to be important for all forms of HFI. Those with social support were less likely to report HFI, with all the odd ratios found to be statistically significant (p < 0.01).

In the econometric models with the household level fixed effects, all the findings remained qualitatively similar. We do not report the results from the household fixed effects as many observations dropped because of the lack of variation within many households; in other words, the estimations happened on a different sample than the ones with or without the village-fixed effects.

### 6.3 HFI and health outcomes

In the second stage of our analysis, in order to understand the association between HFI and health, we ran logistic regression models with three outcome variables—depression, low overall mental health, and low overall health. Given the similarity in the estimates between the parsimonious models and those with the village (and the household level) fixed effects, we report the estimates from bivariate and the parsimonious models with the village fixed effects only. But first we report estimates after adjusting for only predetermined variables in Table 4. The similarity in the estimates from Table 4, Table 5 would clarify the potential role of selection on both observables as well as unobservables through the stability in the size of the estimated coefficients (Altonji et al., 2005).

**Table 4:** Parsimonious multivariate logistic regression: Household food insecurity, depression, overall mental and overall general health in Copán, Honduras.

	Depression			Low Overall Mental Health			Low Overall General Health		
	(1)	(2)	(3)	(1)	(2)	(3)	(1)	(2)	(3)
HFI (1–3)	1.988***	1.769***	1.784***	1.522***	1.354***	1.355***	1.374***	1.188***	1.191***
	(0.037)	(0.034)	(0.036)	(0.027)	(0.026)	(0.027)	(0.024)	(0.023)	(0.024)
Female		1.675***	1.710***		1.407***	1.449***		1.157***	1.190***
		(0.048)	(0.050)		(0.040)	(0.042)		(0.032)	(0.034)
Age		1.030***	1.030***		1.029***	1.030***		1.037***	1.038***
		(0.001)	(0.001)		(0.001)	(0.001)		(0.001)	(0.001)
Indigenous		1.040	1.460***		0.826***	1.054		0.827***	1.164***
		(0.045)	(0.084)		(0.036)	(0.060)		(0.035)	(0.066)
Widowed		1.178**	1.184**		1.015	0.996		0.891	0.888
		(0.091)	(0.092)		(0.074)	(0.073)		(0.067)	(0.067)
Born In the Village		0.888***	0.892***		0.858***	0.885***		0.875***	0.921***
		(0.026)	(0.027)		(0.024)	(0.027)		(0.025)	(0.028)
Constant	0.236***	0.084***		0.330***	0.140***		0.467***	0.182***	
	(0.008)	(0.004)		(0.011)	(0.007)		(0.015)	(0.009)	
									
N	24,760	24,743	24,743	24,760	24,743	24,743	24,760	24,743	24,743
No. of Hhs									
No. of Villages			176			176			176

Note: Robust standard errors clustered at the village level in parentheses; ***p < 0.01, **p < 0.05, *p < 0.1; Column 1s: Odd ratios from an unadjusted logistic regression; Column 2s: Adjusted odd ratios; Column 3s: Adjusted odd ratios after adjusting for village level fixed effects.

**Table 5:** Full specification multivariate logistic regression: Household food insecurity, depression, overall mental and overall general health in Copán, Honduras.

	Depression		Low Overall Mental Health		Low Overall General Health	
	1	2	1	2	1	2
HFI (1–3)	1.988***	1.787***	1.522***	1.355***	1.374***	1.198***
	(0.037)	(0.037)	(0.027)	(0.028)	(0.024)	(0.025)
Female		1.706***		1.404***		1.122***
		(0.053)		(0.043)		(0.034)
Age		1.031***		1.029***		1.038***
		(0.001)		(0.001)		(0.001)
Indigenous		1.458***		1.034		1.141**
		(0.086)		(0.061)		(0.067)
Widowed		1.175**		1.011		0.928
		(0.094)		(0.076)		(0.072)
Plan to Migrate		1.777***		1.276***		1.064
		(0.120)		(0.087)		(0.073)
No Religion		0.901**		0.872***		0.782***
		(0.037)		(0.036)		(0.032)
Both Parents in the Village		0.909***		0.924**		1.009
		(0.033)		(0.033)		(0.036)
No Education		1.096		1.333***		1.115
		(0.110)		(0.139)		(0.112)
Grade 1-6		1.087		1.407***		1.481***
		(0.102)		(0.138)		(0.139)
Some Secondary Education		1.209*		1.438***		1.518***
		(0.129)		(0.159)		(0.161)
Not Married		1.085**		0.942		0.862***
		(0.042)		(0.036)		(0.033)
Born in the Village		0.898***		0.905***		0.929**
		(0.030)		(0.030)		(0.030)
Wealth Index 2 (Poorer)		0.919		0.912*		0.925
		(0.047)		(0.047)		(0.047)
Wealth Index 3		0.944		1.009		0.995
		(0.048)		(0.050)		(0.049)
Wealth Index 4		1.011		0.988		1.026
		(0.050)		(0.048)		(0.050)
Wealth Index 5 (Richest)		0.981		1.010		1.028
		(0.047)		(0.048)		(0.048)
Constant	0.236***		0.330***		0.467***	
	(0.008)		(0.011)		(0.015)	
Observations	24,760	23,680	24,760	23,680	24,760	23,680
Number of Village		176		176		176

Note: Robust standard errors clustered at the village level in parentheses; ***p < 0.01, **p < 0.05, *p < 0.1; Column 1s: Odd ratios from an unadjusted logistic regression; Col 2s: Adjusted odd ratios; Col. 3s: Adjusted odd ratios after adjusting for village level fixed effects.

We used the 3-level HFI variable—No HFI (1), Mild/Moderate HFI (2), and Severe HFI (3). The odd ratios for HFI were in the range of 1.99 to 1.78 with depression as the outcome variable, depending on whether the specification was bivariate or the parsimonious one with just the village fixed effects, see Table 4. On taking the assumptions discussed in the variable selection methods, Table 4 (Col. 3) shows that moving from a food-secure household to severe food security led to 3.57 times greater odds (p < 0.01) of reporting the incidence of depression in any given village. The corresponding numbers for mental health and low overall health were 2.70 and 2.38 times greater odds, respectively, both with p < 0.01. When the sample was restricted to the Copan Ruinas municipality only, the odds ratios were found to be of the same magnitude. In the full model, after adjusting for a constellation of individual-level confounders, the effect size of the estimates remained practically the same (see Table 4, Table 5).

The variable ‘being born in the village’ shows protective effects against poor physical and mental health: the odd ratios range from 0.89 for depression, 0.86 for low overall mental health, and 0.91 for overall health (p < 0.01). A similar pattern holds for the variable ‘both parents of the respondent lived in the same village,’ although the pattern continued to hold only for the two of three health outcomes used in this study: depression and overall mental health. Therefore, living in the vicinity of one's family is associated with better outcomes with respect to depression and mental health in general, but not for the measure of overall health.

The comparison of Table 4, Table 5 suggests that the relationship between HFI and all three measures of health outcomes remained robust across different models—odd ratios associated with HFI continued to remain robust across different regression models.

## 7 Discussion

Earlier studies of HFI conducted in Honduras, Nicaragua, Uganda, and other countries have used smaller samples and focused on specific sub-populations such as pregnant mothers and caregivers. Our findings based on a much larger sample suggest that more individuals reported stress and anxiety about being food insecure than those who actually ran out of food. Therefore, a better resource planning at the household level could potentially play a critical role in reducing the pernicious effects of food-related stress and anxiety—a contributory factor in keeping a family in a nutrition related poverty trap (Fiese et al., 2016).

Individuals who identified themselves as indigenous turned to be at high risk of HFI even after controlling for confounding factors. This points towards a need for designing specific and targeted policies to reduce the incidence and prevalence of food insecurity in the region. After controlling for the village- and household-level fixed effects, the reduction in the magnitude of the estimate on indigenous variable suggests that village and household level policy intervention can potentially be used to ameliorate food insecurity for indigenous population. Keeping that in mind, there is an urgent need to identify the village and household level levers to effectively tackle the problem of food insecurity.

Age turned out to be an important risk factor: older respondents reported both a higher level of food security related stress and also a higher incidence of running out of food. One potential explanation could be a biased intrahousehold allocation of food—favoring both younger cohorts, who are the relatively more vulnerable members of a family. In addition, it is quite likely that those in the age group of 51–65 could be more aware of the access and availability of food in the family as they might have recently been or could currently be the primary bread providers for the household.

Marital status was also found to be a risk factor for HFI. Those reporting not married were found at a lower risk of experiencing any form of HFI: the odds ratio was less than one (0.83) and statistically significant. However, marital status was found associated with a slightly higher odds of being severe HFI. Albeit, this finding was statistically insignificant, it does hints at family as a risk mitigating factors for individuals who are not married, and who are likely to be younger and not having the pressure of taking care of a family. This hypothesis would need to be tested through further research specifically designed to answer this important question.

Affiliation with religious institutions did not protect against food insecurity. However, a higher likelihood of having denser social networks and access to psychological support through family networks could be the reasons that having both parents living in the same village emerged to be a factor protecting against the risk of HFI.

The magnitude of odds ratios for the variable ‘intending to migrate’ suggest that those planning to migrate were more likely to experience HFI. One potential reason can be the savings needed to cover the cost of migration, leading to a diminished budgetary allocation for food. Research uncovering the directionality and nature of association between food insecurity and migration has focused their attention on opposite direction than the one we have highlighted in this paper (Smith & Floro, 2020; Smith & Wesselbaum, 2020). In the context of our findings, we have developed the innovative hypothesis that the intention to migrate and the corresponding coping mechanisms (including saving money to be able to migrate) could itself be a source of the distress leading to food insecurity. To the best of our knowledge, there is no other study that has explored this channel. However, one study from Bangladesh suggests that subsidizing migration to the cities showed promising results in terms of ensuring food security (Meghir et al., 2019). We recommend for future mixed methods research, including longitudinal studies, to focus on answering this important hypothesis.

The risk factors identified above strongly suggest that special attention needs to be paid to alleviating HFI among women, indigenous individuals, older populations, and those planning to migrate. Our findings point to the need to take into account diverse demographic factors to develop well-targeted policies. A qualitative study needs to be conducted to see whether the plans to migrate are pushing people to take the risk of being food insecure. If that is found to be the case, a policy framework that provides subsidies for potential migrants could help mitigate the risk of migration on HFI.

The five common sociodemographic risk factors for HFI mentioned in the literature can be pared down to three in our analyses: education, social support, income. These three variables were all found to be the risk factors for HFI among rural Hondurans. Beyond these variables, we uncovered gender, ethnicity, and intention to migrate also additional risk factors for HFI.

Many of the risk factors discussed earlier experienced change in the magnitude after adjusting for village-level or household-level fixed effects (see Table 3, Table 4A). Thus, one can surmise that the village and household level unobserved factors appeared to be mediating factors for the risk factors identified for the HFI.

Given the consistency of the estimated odds ratios across different econometric models in conjunction with the assumptions built in the variable(s) selection framework (mentioned in Section 5), it looks as if the food insecurity is a form of aggregate risk faced by the Honduran population given its geographic location in the ‘Dry Corridor’ mentioned earlier. This could potentially explain why the adjustment for the village and household level unobservables—on statistical ground—failed to *qualitatively* affect the coefficients in any significant manner (see Table 3, Table 4A).

Given the challenge of using village and household fixed effects mentioned earlier, we suspected that aggregating the effects of all four municipalities could potentially give us a perverse relationship aka Simpson's Paradox (Pearl, 2014). Simpson's paradox occurs when the relationship between treatment and outcome variable for the aggregated data turned out to be opposite to the one in the disaggregated groups in the population. We, thus, conducted the second stage analysis just on those residing in Copan Ruinas municipality (the biggest size municipality in the data), and we found that all the coefficients qualitatively remained the same. The results from this exercise helped in rejecting the possibility that a spurious relationship between HFI and health outcomes.

## 8 Conclusions

HFI, a condition in which individuals and families have poor access to adequate food owing to limited resources, is of critical concern globally. In order to develop cost-effective interventions, it is important first to identity the most vulnerable populations. Among the rural and marginalized population in the Copan Ruinas area of Honduras, females, indigenous population, and those planning to migrate turned out to be more food insecure than the rest.

HFI is a necessary—but not the sufficient factor—to support good health. For this reason, it is important for governments to include food security actions as part of a package that also addresses other determinants of health including access to proper housing, health care, possibly subsidizing the cost of migration.

Additionally, given the number of Hondurans found seeking asylum in the US, it is important, and it is a moral and human right issue now to build a comprehensive framework to understand the factors leading to a life of hardship, food insecurity (including, hunger), and violence for a large swathe of Hondurans. Our findings call for paying special attention to the HFI risk among those planning to emigrate, given the large spatial heterogeneity across villages and municipalities reported earlier. The cost to penalize the migrants apprehended at the U.S. border could have been better utilized in creating opportunity for human flourishing in Honduras itself (Nazario, 2019).

## 9 Limitations and future directions

The major limitation of our work is that we have included only two items out of 14 included in the original scale to measure household level food insecurity. However, as it has been shown in the USA, our two-item scale, that captured two HFI situations representing highly contrasting levels of severity (i.e., being worried vs. actually running out of food) yielded an indicator with strong predictor and convergence validity. From the policy perspective, one of the concerns is that this study is based on a cross-sectional analysis. In addition, HFI is not just about food availability and affordability, but also about cultural norms about socially acceptable means of acquiring food and knowledge about nutrition, and we were unable to directly address this aspect of HFI in this paper (Jones, 2017); however, adjusting for ethinity and village-level fixed effects do control for this feature to some extent.

Given that food insecure individuals and households are more likely to suffer from micronutrient deficiencies, they are more likely to suffer from cognitive and non-cognitive skill deficiencies leading to sub-optimal health behaviors, further accentuating food insecurity. The data limitation did not allow us to probe the mechanisms through which HFI impact health outcomes. In this paper, given the cross-sectional nature of the analyses we were unable to explore the role that the duration of food insecurity—a crucial aspect of HFI—plays in mental and physical health deterioration.
